# Design and Delivery of a Tailored Intervention to Implement Recommendations for Multimorbid Patients Receiving Polypharmacy into Primary Care Practices

**DOI:** 10.1155/2015/938069

**Published:** 2015-01-05

**Authors:** Cornelia Jäger, Joachim Szecsenyi, Jost Steinhäuser

**Affiliations:** Department of General Practice and Health Services Research, University Hospital Heidelberg, Voßstraße 2, 69115 Heidelberg, Germany

## Abstract

*Introduction.* Managing polypharmacy is particularly demanding for general practitioners as coordinators of care. Recently, a German guideline for polypharmacy in primary care has been published. This paper describes the content and delivery of a tailored intervention, which aims at improving the implementation of guideline recommendations for polypharmacy into practice, considering individual barriers. *Materials and Methods.* Firstly, barriers for implementation and the corresponding strategies to address them have been identified. On this basis, an intervention consisting of a workshop for health care professionals and educational materials for patients has been developed. The workshop focused on knowledge, awareness, and skills. The educational materials included a tablet computer. Practice teams will elaborate individual concepts of how to implement the recommendations into their practice. The workshop has been evaluated by the participants by means of a questionnaire. *Results.* During the workshop 41 possible sources of medication errors and 41 strategies to improve medication management have been identified. Participants evaluated the workshop overall positively, certifying its relevancy to practice. *Discussion.* The concept of the workshop seemed appropriate to impart knowledge about medication management to the participants. It will have to be evaluated, if the intervention finally resulted in an improved implementation of the guideline recommendations.

## 1. Introduction

There are an increasing number of patients suffering from multiple chronic conditions and receiving polypharmacy [1]. Although a uniform definition and objective terminology are still lacking, polypharmacy is commonly defined as permanent intake of five or more drugs [2, 3]. It is well known that this patient group has a higher risk of potentially avoidable and potentially harmful adverse drug reactions (ADR) [4].

Managing multimorbid patients with polypharmacy is particularly demanding in primary care, as it requires coordination of multiple prescribers, profound pharmacological knowledge, and intense monitoring of patients [5]. As Germany is a representative of countries without an established gate-keeping system and with a strong ambulatory specialist care system in addition to primary care and hospital care, this challenge becomes even more evident. Patients in Germany have free choice of doctors and do not have to be registered at any primary care practice. Therefore different care providers are able to alter medication regimens without communication with the general practitioner (GP) [6]. To date there is no established patient tracking system in Germany and physicians' awareness of the impact of such interface problems seems to be low [7].

**Figure 1:**
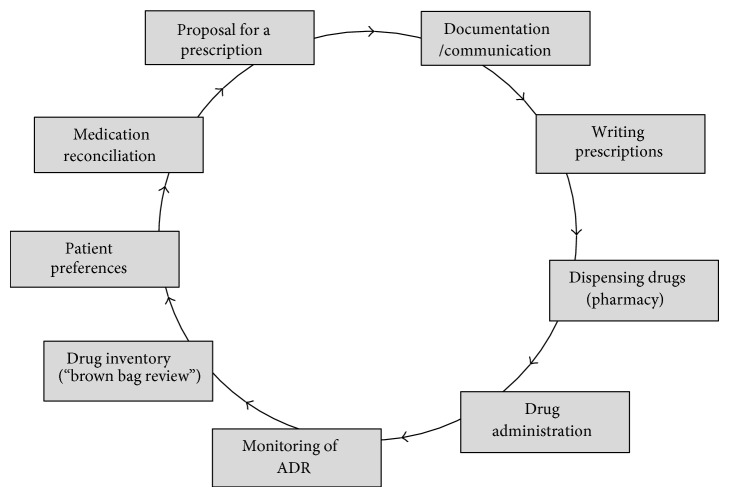
Medication process as depicted in a German guideline for multimedication [8], modified and translated into English.

Recently, a German guideline for the management of polypharmacy in adult and geriatric patients in primary care has been published [8]. This guideline is oriented on the medication process as suggested by Bain et al. [9] (see Figure 1) and contains amongst others three recommendations.

*(i) Structured Medication Counselling (SMC)*. Patients with polypharmacy and additional risk factors for medication problems should receive SMC at least once per year. SMC comprises a complete inventory of the actually taken medication (so called “brown bag review”) and the assessment of patient adherence and possible application problems. A separate appointment should be planned for SMC [8, 10].

*(ii) Consequent Use of Medication Lists*. All patients with polypharmacy should take along an updated and complete medication list. The minimum standards of a comprehensible medication list have previously been specified by the Drug Commission of the German Medical Association [8, 11].

*(iii) Medication Reviews to Reduce Potentially Inappropriate Medication (PIM)*. Appropriateness of medication can be defined by explicit criteria (usually “drug-to-avoid-lists”) and implicit criteria (usually checklists). It is recommended that physicians regularly review the medication regimens of patients with polypharmacy with the aid of checklists, such as the “medication appropriateness index (MAI)” [12] and/or drug-to-avoid lists, such as the “PRISCUS-list” [13], a list similar to the Beers-criteria [14] adapted to the German context.

Performance gaps concerning medication management have been reported by a set of international studies and current research in Germany reveals that to date these recommendations have not been well implemented in German routine care. Although there is evidence that SMC may increase patient satisfaction [15], improve adherence, and reduce ADR and hospitalizations [16], there is no established structure for medication counselling in Germany. Patients criticize the lack of information about possible side effects and feel that there is little room to discuss their concerns during the consultation [17]. Discrepancies between the medication documented on the medication lists and the actually taken medication are frequent. Bad management (e.g., medication list not updated) is a frequent cause for such discrepancies [18]. A considerable proportion of patients receive medications, which are not indicated or not evidence-based [19].

As amplified below, an increasing number of intervention studies evaluating approaches to improve the appropriate use of polypharmacy are being reported. However, the informative value of such studies is frequently low due to poor designs and insufficient provision of details on the content, development and delivery of the interventions, which makes it difficult to reproduce them [20]. Implementation research suggests that implementation programs should be tailored to individual barriers to introduce evidence-based knowledge into practice [21, 22]. This study is part of the “tailoring interventions for chronic diseases (TICD)” project [23], in which a four-step approach for tailoring is used [24]. In the context of this project we have developed a tailored intervention which aims at improving the implementation of the above mentioned recommendations into German primary care practices. The “polypharmacy in multimorbid patients study (PomP)” evaluates the effect of this implementation program in a cluster-randomized controlled trial [25]. The aim of this paper is to comprehensively describe the content and delivery of the implementation program, so that others could reproduce and advance it.

## 2. Materials and Methods

### 2.1. Development of a Tailored Implementation Intervention

We used qualitative approaches (group discussions, interviews) targeted at health care professionals and patients to identify barriers for the implementation of the named recommendations and—in a second step—strategies to address these barriers. This second step also included a summary of the current literature on interventions aiming at improvement in the three fields. The summary was the result of a selective literature research and is depicted in Table 1. It was presented to the interviewees as a stimulus to create new ideas or to select interventions they favored.

We used the criteria “relevance” and “modifiability” to prioritize the identified barriers and the criteria “assumed impact” and “feasibility” to prioritize the identified strategies by points scores. Based on this ranking, we designed an implementation intervention, in which specific strategies were selected in order to modify specific barriers [24].  Table 2 lists the selected barriers and strategies.

### 2.2. Target Group of the Intervention

The intervention of the PomP study targeted primary care physicians, who were enrolled in a GP-centred care contract of a large German health insurance and organized in quality circles [25]. As part of this voluntary program, physicians receive feedback on their prescribing behaviour based on administrative data [26]. Therefore the PomP intervention has to be seen as an add-on-intervention. Furthermore patients aged >50 years, suffering from at least 3 chronic conditions and being prescribed more than 4 drugs permanently, were targeted [25].

### 2.3. Design of the Implementation Intervention

The implementation intervention basically consisted of three components: the elaboration of (a) individual practice concepts, (b) training for health care professionals, and (c) educational materials for patients.

*(a) Training for Health Care Professionals*. Target groups for the training were the general practitioners (GPs) and health care assistants (HCAs) of the intervention practices. The training followed a constructivistic approach, including the activation of existing knowledge, the adaption of differences in existing knowledge via short input presentations, and the exchange of experiences. The format of a “workshop” was chosen to address different prerequisites for behaviour change, which are awareness, knowledge, resources, and skills.

*(i) Knowledge*. Topics covered by the input presentations were the prevalence and consequences of polypharmacy, the recommendations given by the guideline and performance gaps in German primary care regarding these recommendations.

*(ii) Awareness*. An analysis of possible sources of medication errors on the different levels of the medication process (Figure 1) and identification of strategies to avoid these errors was done by brainstorming in small groups using a card technique.

*(iii) Resources*. Tools to facilitate medication reviews and counselling were given to the health care professionals. A checklist consisting of modified items of the medication appropriateness index and two additional items referring to the QT-interval and the “PRISCUS list” was provided to structure medication reviews. Additionally each GP received a tablet computer, on which several free online resources facilitating medication reviewing were compiled, for example, search engines for QT-drugs [27], nephrotoxic drugs [28], or free software for interaction checks [29], since such tools are frequently not default components of the practice-software in Germany. Furthermore checklist for medication counseling, “brown bag reviewing” as well as a comprehensive medication list template (which meets the minimal requirements as defined by the Drug Commission of the German Medical Association) [11] was made available.

*(iv) Skills*.  Different case study exercises for GPs and HCAs were conducted. GPs evaluated the complex medication regime of a fictitious patient with the aid of the checklist and the online resources. HCAs performed a “brown bag review” on a fictitious patient in a “role-play” scenario using the checklist and the bags provided, complete with the actual (but empty) medication packages.

*(b) Educational Materials for Patients*. The practices received several materials to increase the self-management-abilities of patients regarding medication related issues.

**Figure 2:**
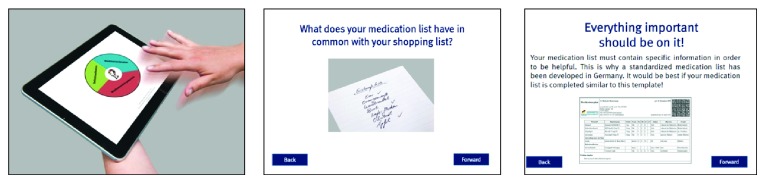
A tablet based information tool for patients.

*Information Tool on a Tablet Computer*. The tablet computers handed out to the health care professionals contained an interactive information tool for patients, covering the topics “correct use of medication lists,” “medication counselling,” and “over-the-counter drugs.” To address language barriers, the tool was—beside German—also available in English and Turkish, as there are a high number of patients with migration background, especially from Turkey, in Germany. The format was a simple, bidirectional website with only forward and back buttons using mainly pictures and a humorous language to convey the messages. After clicking through the entire website, patients could do a short multiple-choice quiz. Physicians were encouraged to let patients complete the information tool during the SMC.  Figure 2 shows the tablet based information tool.

*Posters and Reminders*. Posters encouraging patients to always bring along their medication lists and paper bags with an imprint reminding patients to bring their medication packages to the counseling interview were provided.

*(c) Elaboration of Tailored Practice Concepts*. At the end of the workshop all practice teams were asked to elaborate individual concepts tailored to the specific circumstances in their practice to implement the guideline recommendations into routine care. They were asked to send this concept in written form to the study center within the next two weeks. For this purpose, the possible sources of errors and solution strategies gathered during the group work were sent to the participants in hard copy.

### 2.4. Evaluation

At the end of the workshop all participants completed a piloted and validated questionnaire [30] consisting of 10 items reflecting different quality indicators for continuous medical education such as “content,” “participation,” or “organization.” The survey was analyzed descriptively merging the response categories “content” and “rather content” as well as “uncontent” and “rather uncontent”.

## 3. Results

### 3.1. Participants and Setting

12 GPs and 8 HCAs from 8 practices participated in the workshop. The workshop was held in February 2014 and lasted for 4 hours and took place in a seminar room of a hospital located in the surrounding area (less than 100 km) of the practices.  Table 3 shows the characteristics of the participants. The age and gender patterns are comparable to the results of a larger survey among physicians in Germany [31].

### 3.2. Results of the Group Work

Table 5 shows the results of the analysis of possible sources of errors and strategies to avoid them on the various levels of the medication process. In total 41 sources of errors and 41 strategies were found.

### 3.3. Results of the Evaluation

14 of the 20 participants completed the evaluation questionnaire. As depicted in Table 4 the majority of the participants evaluated the workshop overall positively. 93% were content with the practical relevance and 79% stated that the participation in the training was overall worthwhile.

## 4. Discussion

This paper focuses on the design and delivery of a tailored intervention. The PomP study examines an implementation program, which aims at improving the implementation of recommendations for polypharmacy into primary care practices. The implementation program consisted of two main components: a workshop for GPs and HCAs and educational materials for patients. On the basis of the results of the workshops, practice teams will elaborate individual concepts of how to implement the recommendation into their practice.

The positive evaluation by the participants and the high number of barriers and strategies gathered during the group work indicate that the format of the workshop is appropriate to sensitize health care professional for optimized medication management in primary care.

According to specific frameworks for chronic illness care, such as the chronic care model, patient self-management is one crucial column in the care of the chronically ill [32]. Therefore several components of our intervention intended to strengthen patients' self-management abilities. While posters are a frequently used strategy with rather low impact on behaviour change [33], the tablet-based information tool for patients is an innovative approach. It could be argued that the use of such modern technology is not appropriate for the target group of elderly, multimorbid patients. Yet there is evidence that tablet computers can be efficiently used in the treatment of patients suffering from an early-stage dementia and studies exploring further uses of this technology in patient care have been requested [34]. The assessment of the usability and influence of the tablet computers on patient behavior will be part of the process evaluation of the main study [35]. If this technology proves to be useful for the target group of elderly patients, more advanced interactive training tools following the serious gaming approach could be developed [36].

This intervention was developed as a tailored intervention, meaning that barriers and strategies for the implementation of evidence-based recommendations have been identified previously to the design of the intervention. In addition to this tailoring process in the developmental phase, strategies to avoid errors for each step of the medication process were found. These strategies will be used to elaborate individual practice concepts as a further step of tailoring in the delivery phase of the intervention [24].

Comparing the components of our intervention with interventions in earlier studies (Table 1), it becomes apparent that frequently used strategies (such as training or reminders) as well as innovative approaches (such as individualized practice concepts and tablet-based information material) were used. The latter could be ascribed to the conducted tailoring. It will have to be evaluated in future studies whether the intervention actually increased the implementation of the guideline recommendations into practice and to what extent the tailoring process contributed to this.

## Figures and Tables

**Table 1:** Summary of the current literature on interventions aiming at an improved management of polypharmacy as presented to the interviewees.

Recommendation/implementation objective	Interventions/intervention components	References
Reduce potentially inappropriate medication	(i) Involve pharmacists into medication checks (ii) Multidisciplinary case conferences (iii) Consultation of geriatricians before hospital discharge (iv) Computer-assisted medication checks (v) Training of patients on ADR and adherence (vi) Training of doctors and nurses on ADR, guidelines, and alternative drugs	[20, 37–44]

Consequent use of medication lists	(i) Reminders (letters, phone calls, and “medication bags”) for patients to bring along their medication and medication/medication list (ii) Training of patients on drug safety (iii) Training of doctors on medication reconciliation (iv) Delegate “Brown Bag Reviews” to HCAs (v) Feedback to physicians on the accuracy of the medication lists in their practice (vi) Web portal, where patients could enter their medication and receive emails reminding them to update their medication list. Physician received emails if patients made changes (vii) Medication reconciliation by pharmacists using various sources (e.g., prescription data or discharge letters)	[45–49]

Structured medication counselling	(i) Feedback reports to physicians on patients' satisfaction with information about medicine (ii) Electronic messages sent to patients 10 days after medication alterations with questions about adherence and ADR (iii) Individualized patient information leaflets about their medication	[15, 37, 50]

ADR = adverse drug reactions, HCA = health care assistant.

**Table 2:** Strategies used and barriers intended to be modified by the implementation intervention.

Barriers	Strategies
Lacking expert knowledge on medication management Low feasibility of checklists for medication reviews	Training

Medication counselling is not routine Difficulties to define the target group for medication counselling	Individual practice concepts Provision of a checklist

Medication lists are not available at interfaces Lacking self-management abilities of patients Language barrier	Educational materials for patients

Lacking standardization of medication lists	Template of a medication list

**Table 3:** Characteristics of the workshop participants.

	GPs	HCAs
Number	*n* = 12	*n* = 8
Mean age (years)	53 (40–63)	29,1 (25–45)
Sex	Male: 67% (*n* = 8) Female: 33% (*n* = 4)	100% female
Mean work experience (years)	18 (4–30)	9,4 (3–22)

**Table 4:** Evaluation of the workshop by the participants.

	Number	(Rather) content	Partly content, partly uncontent	(Rather ) uncontent
Information content	*n* (%)	13 (92,9)	7,1 (1)	0
Presentation	*n* (%)	14 (100)	0	0
Participation	*n* (%)	13 (92,9)	7,1 (1)	0
Work climate	*n* (%)	14 (100)	0	0
Practical relevance	*n* (%)	13 (92,9)	0	1 (7,1)
Organisation	*n* (%)	14 (100)	0	0
Materials	*n* (%)	14 (100)	0	0
Exchange with colleagues	*n* (%)	14 (100)	0	0

	Number	Yes	Do not know	No

Have you received new impulses for the management of multimorbid patients receiving polypharmacy in your practice?	*n* (%)	13 (92,9)	1 (7,1)	0
Has the workshop overall been worth your while?	*n* (%)	11 (78,6)	2 (14,3)	1 (7,1)

**Table 5:** Results of the group work during the workshop.

Medication process	Sources of error	Strategies to avoid errors
(1) Prescription suggestions	(i) False dose (kidney function!) (ii) Drug not indicated (any more) (iii) Dangerous interactions (iv) False application (e.g., halving slow release tablets, grinding enteric-coated tablets, etc.) (v) Doubling prescriptions (vi) PIM-prescriptions	See (9)

(2) Documentation	(i) No medication plan issued (ii) Not updating the medication plan, for example, between services (admissions and transfers) (iii) Plan not available/patients do not have the plan with them (iv) Transcription errors (v) Poor legibility (vi) No documentation when issuing drug samples	(i) Tracking (patient lists for admissions and transfers) (ii) Encouraging patient self-responsibility and self-management (iii) Document issuing of sample packages

(3) Writing prescriptions	(i) Not considering repeat prescriptions (ii) Dose error (iii) Dose not recorded on the prescription (iv) Software error or PC user error on (e.g., switches column)	(i) Telephone prescriptions/no routine filling of prescriptions (ii) Update and check medication plan with every prescription → (iii) Patient education: no filling of prescriptions without medication list (iv) Checking prescription timeframe on every repeat prescription (v) Thorough checking of prescription requests from nursing homes (vi) Education of HCA about high risk medication

(4) Dispensing medications	(i) Mixing up brand names and generic names/discount contracts (ii) Dangerous self-medication (iii) Issuing medication without prescription/later presentation of prescription (iv) Issuing of incorrect medication or incorrect dose strength	(i) Medication lists specifying the active ingredients instead of trade names. (ii) Checking with pharmacies in the area (iii) Advising patients to use a regular pharmacy (own choice) (iv) Checking interactions in the pharmacy (v) Using blister packs (vi) “Aut idem”-prescriptions of risk medications (e.g., Marcumar, L-Thyroxin)

(5) Administration of medications	(i) Unintended nonadherence (forgotten to take medication) (ii) Intentional avoidance/dose reduction or self-determined dose skipping (iii) Problems with administration: swallowing, dividing tablets (phenprocoumon!), drops, inhalers, injections, patches (iv) Unintended intake of double dose due to generic drugs/various brand names (v) Confusing medications (vi) Daily intake of preparations that are intended for once a week (e.g., vitamin D, Iodine, biphosphonate, methotrexate)	(i) Use combination drugs (ii) Avoid halving doses (iii) Intake every morning (iv) Patient education (e.g., Education video for Marcumar patients from Göttingen Uni) (v) Reminder for patients (vi) Checking administration by HCA (vii) “Money Counting Test” (viii) Administration information from the pharmacy (ix) Support from relatives and nursing stuff

(6) Monitoring of ADR	(i) “Prescription cascade” (ii) Repeat prescription despite a lack of improvement (iii) Lack of/infrequent creatinine levels control (iv) Lack of/infrequent INR control (v) Lack of/infrequent blood sugar levels control	(i) Planned withdrawal trials (ii) Case-Management (iii) Pharmacovigilance (iv) Prescription of risk medications bound with monitoring requirements (e.g., ECG with repeat prescriptions of certain antidepressant agents)

(7) Stocktaking/inventory	(i) Lack of/incomplete assessment of self-medications (ii) Lack of/incomplete assessment of prescriptions from other doctors (iii) Medication from the partner taken or brought in	(i) Appointment for systematic review of medications (“Brown Bag Review”) (ii) Reminder before appointment for patient to bring all their medications with them

(8) Patient preferences	(i) Patient insisting on/or declining a particular medication (ii) Subjective view of intolerance (iii) Problems with understanding due to poor education level, low intelligence, or language barriers	(i) Do not assume a medication preference/directly question patient if they have medication preferences (ii) Relationship management so that admitting nonadherence is possible (iii) Discuss fears/illness concept with the patient (iv) Shared decision making (v) Documentation when medications are declined (vi) Prescription of risk medication on nonsubsidised forms (patient carries cost, e.g., for sleeping tablets, NSAIDs)

(9) Medication reconciliation	(i) Complete overview of medications is unknown due to prescriptions from various doctors and over-the-counter medications (ii) Lack of specialist knowledge (iii) Uncertainty regarding discontinuation of medications prescribed by specialist or clinics (iv) Conflicting guidelines	(i) Online reference resources PRISCUS-List (ii) Medication appropriateness Index (MAI) for systematic review of medications (iii) Software for checking interactions (iv) Binding “Disease Management Programs” with medication checks
